# The Sec7 Guanine Nucleotide Exchange Factor GBF1 Regulates Membrane Recruitment of BIG1 and BIG2 Guanine Nucleotide Exchange Factors to the *Trans*-Golgi Network (TGN)*

**DOI:** 10.1074/jbc.M112.438481

**Published:** 2013-02-05

**Authors:** Jason Lowery, Tomasz Szul, Melanie Styers, Zoe Holloway, Viola Oorschot, Judith Klumperman, Elizabeth Sztul

**Affiliations:** From the ‡ Department of Cell, Developmental, and Integrative Biology, University of Alabama at Birmingham, Birmingham, Alabama 35294,; the § Department of Biology, Birmingham Southern College, Birmingham, Alabama 35254,; ¶ Wellcome Trust Centre for Human Genetics, University of Oxford, Oxford OX3 7BN, United Kingdom, and; the ‖ Department of Cell Biology, University Medical Center Utrecht, 3584CX Utrecht, The Netherlands

**Keywords:** ARF, Golgi, Guanine Nucleotide Exchange Factor (GEF), Membrane Trafficking, Vesicles, COPI

## Abstract

Three Sec7 guanine nucleotide exchange factors (GEFs) activate ADP-ribosylation factors (ARFs) to facilitate coating of transport vesicles within the secretory and endosomal pathways. GBF1 recruits COPI to pre-Golgi and Golgi compartments, whereas BIG1 and BIG2 recruit AP1 and GGA clathrin adaptors to the *trans*-Golgi network (TGN) and endosomes. Here, we report a functional cascade between these GEFs by showing that GBF1-activated ARFs (ARF4 and ARF5, but not ARF3) facilitate BIG1 and BIG2 recruitment to the TGN. We localize GBF1 ultrastructurally to the pre-Golgi, the Golgi, and also the TGN. Our findings suggest a model in which GBF1 localized within pre-Golgi and Golgi compartments mediates ARF activation to facilitate recruitment of COPI to membranes, whereas GBF1 localized at the TGN mediates ARF activation that leads to the recruitment of BIG1 and BIG2 to the TGN. Membrane-associated BIG1/2 then activates ARFs that recruit clathrin adaptors. In this cascade, an early acting GEF (GBF1) activates ARFs that mediate recruitment of late acting GEFs (BIG1/2) to coordinate coating events within the pre-Golgi/Golgi/TGN continuum. Such coordination may optimize the efficiency and/or selectivity of cargo trafficking through the compartments of the secretory pathway.

## Teaser

**Background:** GBF1, BIG1, and BIG2 are guanine nucleotide exchange factors (GEFs) that activate ARFs to regulate secretory traffic.

**Results:** GBF1 activity promotes subsequent recruitment of BIG1/2.

**Conclusion:** The three GEFs are functionally coupled in a GEF/ARF/GEF cascade.

**Significance:** Coupling integrates all coating events within the pathway to simultaneously regulate passage of cargo through multiple compartments.

## Introduction

ADP-ribosylation factor (ARF)^3^-activating guanine nucleotide exchange factors (ARFGEFs) have been shown to play a key role in the biogenesis and maintenance of the secretory pathway. Treatment of cells with the drug brefeldin A (BFA), an inhibitor of the large ARF GEFs GBF1, BIG1, and BIG2 results in the disassembly of the secretory pathway (1–3). The key enzymatic function of ARFGEFs is to catalyze the exchange of GDP for GTP on ARFs, leading to ARF activation (4). Like all small GTPases, ARFs cycle between a GDP-bound inactive cytosolic form and a GTP-bound activated form that associates with membranes and can support membrane trafficking. GEF-mediated activation is required for ARFs to facilitate recruitment of coat proteins, including coat protein complex I (COPI) and the clathrin adaptor proteins AP1, AP3, and AP4 and the Golgi-localized γ-ear-containing ARF-binding proteins (GGAs) (5–8). The ARFs and coats function together to facilitate sequestration of protein cargoes into newly formed vesicles, allowing for cargo transfer between membrane compartments.

The recruitment of the heptameric COPI coat complex has been well characterized and appears to be regulated by ARF activation mediated by GBF1, a GEF that co-localizes extensively with COPI (9). COPI is recruited to compartments of the early secretory pathway including endoplasmic reticulum (ER) exit sites, the ER-Golgi intermediate compartment (ERGIC), and the Golgi (9–12). RNA interference-mediated depletion studies indicate that GBF1 is the sole GEF responsible for COPI recruitment to all cellular membranes (13–15). Recruitment of COPI occurs through a direct interaction with ARF and facilitates trafficking of membrane proteins between compartments of the early secretory pathway.

ARF activation is also required for the recruitment of the tetrameric adaptor protein 1 (AP1) complex and three monomeric GGA1–3 to the TGN to mediate clathrin recruitment (16). AP1 and the GGAs have been shown to interact in a phosphorylation-dependent manner and to mediate trafficking of the mannose 6-phosphate receptor (M6PR) between the TGN and early endosomes (17–19). GGA1–3 appear to function as a single complex at the TGN, and knockdown of any single GGA by RNA interference affects the expression of the other GGAs and disrupts TGN morphology (20).

The closely related ARFGEFs BIG1 and BIG2 localize to the TGN and endosomal compartments and appear to regulate coating events therein. Double (but not single) depletion of BIG1 and BIG2 inhibits recruitment of AP1 and GGAs to TGN membranes, suggesting that BIG1 and BIG2 may either work together to mediate AP1 and GGA recruitment or that the BIGs can compensate for one another (14, 15). Both BIG1 and BIG2 exhibit partial co-localization with clathrin and clathrin adaptors; BIG1 has been localized to the Golgi/TGN by partial co-localization with mannosidase II (21), TGN38 (a marker of the TGN), and clathrin (9), whereas BIG2 has been localized to the Golgi and TGN regions through co-localization with the γ-adaptin subunit of AP1, GGA3, and M6PR (22). In addition, BIG2 has also been shown to localize to transferrin-containing recycling endosomes (23, 24).

Together, these observations suggest that GBF1 regulates COPI-coating events within the early secretory pathway, whereas BIG1 and BIG2 regulate clathrin-dependent coating at the TGN and endosomal compartments. However, it remains unknown whether these coating events are interdependent and whether the three GEFs cooperate to facilitate the biogenesis of the secretory pathway and cargo progression along the secretory pathway. In this study we investigated the role of GBF1 in regulating coating events at both the early and late secretory compartments. We found that GBF1 is required for recruitment of both the COPI coat to early secretory compartments and of the clathrin-dependent coats (AP1 and GGA2–3) to late secretory compartments. Because of the previously reported roles of BIGs at the TGN, we explored the possibility that GBF1 may regulate recruitment of clathrin adaptors by influencing the localization of BIG1 and BIG2. Interestingly, we found that RNAi-mediated depletion of GBF1 or inactivation of GBF1 (by introducing the E794K inactivating mutation or treating cells with the GBF1-selective drug golgicide (GCA)) inhibited membrane recruitment of BIG1 and BIG2. Importantly, BIG1/2 recruitment was restored in cells treated with GCA by overexpressing the active forms of ARF4 and ARF5. This suggested that GBF1-mediated activation of ARFs may facilitate BIG1/2 association with TGN membranes. In support, we show that GBF1 is present on TGN membranes (in addition to its previously described localization to early secretory compartments) and thus can act as the source of active ARF4 and ARF5 at the TGN. We also show that active forms of ARF4 and ARF5 co-precipitate with BIG2 and that this interaction is mediated by the N-terminal region of BIG2, the same region that is sufficient to recruit BIG2 to TGN membranes *in vivo* (9). Thus, GBF1 appears to act as a master regulator of coating events within the secretory pathway by facilitating both the recruitment of COPI to pre-Golgi and Golgi and the recruitment of BIG1/2 that subsequently activates ARFs to recruit clathrin-dependent coats at the TGN. Such action by the early acting GBF1 may coordinately regulate multiple coating events and allow the optimal spatiotemporal regulation of cargo trafficking through the sequential compartments of the secretory pathway.

## EXPERIMENTAL PROCEDURES

### 

#### 

##### Antibodies, Reagents, and Plasmids

Rabbit polyclonal anti-GBF1 antibodies have been described (11). Mouse monoclonal anti-BIG1 and anti-BIG2 antibodies were made at The Epitope Recognition and Immunoreagent Core Facility (University of Alabama at Birmingham, Birmingham, AL). The following commercially available antibodies were used: monoclonal anti-Golgin-97 from Molecular Probes (Eugene, OR), polyclonal anti-BIG1 from Santa Cruz Biotechnology, Inc. (Santa Cruz, CA), polyclonal anti-calnexin from StressGen (Victoria, Canada), monoclonal anti-AP1 from Sigma, monoclonal anti-EEA1, monoclonal anti-Golgin-245, monoclonal anti-GGA3, monoclonal anti-GGA2, monoclonal anti-MNK, and monoclonal anti-GBF1 from BD Transduction Laboratories (Mississauga, ON), monoclonal anti-AP2 and polyclonal anti-calreticulin from Affinity Bioreagents (Golden, CO), polyclonal and monoclonal anti-GFP from Abcam (Cambridge, MA), monoclonal anti-clathrin heavy chain from Transduction Laboratories (Lexington, KY), polyclonal anti-β-COP and monoclonal anti-GM130 from Affinity Bioreagents, monoclonal anti-HA from Roche Applied Science, and polyclonal anti-TGN46 from Serotec (Oxford, UK). Secondary antibodies conjugated with HRP, Alexa 488, or Alexa 594 were from Molecular Probes (Eugene, OR). BFA and nocodazole (NO) were from Sigma. siLentFect Lipid transfection reagent was obtained from Bio-Rad. GCA was a generous gift from Dr. David B Haslam (Dept. of Pediatrics, Washington University School of Medicine, St. Louis, MO). For immunogold labeling we used Protein A conjugated to 10- or 15-nm gold particles (Cell Microscopy Center, UMC Utrecht, The Netherlands). A rabbit polyclonal antibody against mouse immunoglobulins (DAKO, Heverlee, Belgium) was used as a bridging antibody between mouse monoclonal antibodies and Protein A-gold (Cell Microscopy Center).

The GBF1 cDNA used in this study has been described previously (García-Mata *et al.* (11)). GFP-tagged wild-type GBF1 was constructed by subcloning GBF1 into the pEGFP vector using XhoI and XmaI restriction enzymes. This results in a GFP extension at the N terminus of GBF1. The GFP-tagged GBF1/E794K construct has been described previously (García-Mata *et al.* (11)). The Construct encoding ARF1-Q71I-HA was a generous gift from Dr. Julie Donaldson (National Institutes of Health, Bethesda, MD); Arf4-Q71I-HA was made in our laboratory utilizing mutagenesis protocol based on the wild-type Arf4 obtained from Dr. Julie Donaldson; Arf3-Q71L-HA was a generous gift from Dr. Rick Khan (Emory, Atlanta, GA); Arf5-Q71I-HA was made in our laboratory utilizing mutagenesis protocol based on the wild-type Arf5 obtained from Dr. Sharon Tooze (Cancer Research Institute, London, UK).

##### Cell Culture and Transfection

HeLa cells were grown in minimum essential medium and Dulbecco's modified Eagle's medium supplemented with glucose and glutamine (Mediatech, Inc., Comprehensive Cancer Center, University of Alabama, Birmingham, AL), respectively. Media were supplemented with 10% fetal bovine serum (FBS, Invitrogen), 100 units/ml penicillin and 100 mg/ml streptomycin (Invitrogen), and 1 mm sodium pyruvate. Cells were grown at 37 °C in 5% CO_2_ in 6-well dishes till ∼70% confluence and transfected using Mirus TransIT-LT1 Transfection Reagent (Mirus Bio Corp., Madison, WI) according to the manufacturer's protocol.

##### siRNA and Drug Treatments

siRNAs against human GBF1 (5′-CGAAAUGCCCGAUGGAGCAtt-3′), human BIG1 (5′-CCUCAACUUAGAUAUUUGCtt-3′), and human BIG2 (5′-GCAAACCAACAACUCCCGAtt-3′) were designed, synthesized as annealed primer, and validated by Ambion (Austin, TX). Ambion nontargeting siRNAs were used as negative controls (scrambled). HeLa cells were transfected with siRNA using siLentFect Lipid (Bio-Rad) reagent according to the manufacturer's instructions.

In some cases cells were treated with 5 μg/ml BFA for 1 h, 1 μg/ml GCA for 1 h, or 1 μg/ml NO for 1 h. In cases where a combination of NO and BFA or GCA was used, cells were first treated with NO and then with a mixture of NO and BFA or GCA at previously indicated concentrations.

##### Immunofluorescence and Immunogold Microscopy

For immunofluorescence, HeLa cells were washed in phosphate-buffered saline (PBS), fixed in 3% paraformaldehyde for 10 min, and quenched with 10 mm ammonium chloride. Cells were permeabilized with 0.1% Triton X-100 in PBS. The coverslips were then washed with PBS and blocked in PBS, 2.5% goat serum, 0.2% Tween 20 for 5 min followed by blocking in PBS, 0.4% fish skin gelatin, and 0.2% Tween 20. Cells were incubated with primary antibody for 1 h at room temperature. Coverslips were washed with PBS, 0.2% Tween 20 and incubated with secondary antibodies for 45 min. Coverslips were washed as described above and mounted on slides in 9:1 glycerol/PBS with 0.1% *p*-phenylenediamine (Sigma). Fluorescence patterns were visualized with a Leitz Orthoplan microscope with epifluorescence and Hoffman Modulation Contrast optics from Chroma Technology, Inc. (Brattleboro, VT). Images were captured with a CCD high resolution camera from Roper Scientific (Tucson, AZ) equipped with a camera/computer interface and analyzed using IPLab Spectrum software (Scanalytics Inc., Fairfax, VA).

For immunogold EM, NRK cells were fixed by adding freshly prepared 4% w/v paraformaldehyde (Polysciences) in 0.1 m phosphate buffer (pH 7.4) to an equal volume of culture medium for 5 min followed by post-fixation in 4% w/v paraformaldehyde at 4 °C overnight. Ultrathin cryosectioning and immunogold labeling were performed as previously described (65). In brief, fixed cells were washed with PBS containing 0.05 m glycine, scraped gently from the dish in PBS containing 1% gelatin, and pelleted in 12% gelatin in PBS. The cell pellet was then solidified on ice and cut into small blocks. For cryoprotection, blocks were infiltrated overnight at 4 °C with 2.3 m sucrose. Then they were mounted on aluminum pins and frozen in liquid nitrogen. Ultrathin cryosections were prepared and picked up in a 1:1 mixture of 2.3 m sucrose and 1.8% methylcellulose.

Quantitation of GBF1 distribution was done by collecting data from 6 different EM grids; 3 that were double-immunogold labeled for GM130 (15-nm gold) and GBF1 (APE10 antibody; 10-nm gold) and the other 3 for clathrin (15-nm gold) and GBF1 (10-nm gold). This approach was taken to avoid any bias toward sampling for the *cis* or *trans* side of the Golgi. Grids were scanned for GM130 or clathrin labeling. Sites of positive labeling were checked for the presence of Golgi elements and for GBF1 labeling. If both conditions were met, the Golgi was taken into account, and the distribution of GBF1 within pre-Golgi, different cisternae of the Golgi, and the TGN was counted. Per grid, 10 Golgi bodies were pooled. Percentages of total GBF1 gold particles found over the distinct regions of the Golgi were calculated by determining per grid the relative distribution of the gold (based on sampling of 10 Golgi bodies). These percentages were added up, and the average percentage ± S.E. was calculated.

##### Cell Fractionation

Cells were washed with PBS and disrupted in 300 ml of homogenization buffer (50 mm HEPES-KOH (pH 7.5), 100 mm KCl, 1 mm MgCl_2_, 1 mm DTT) containing Protease Inhibitor Mixture Tablets, EDTA-free (Santa Cruz Biotechnology, Santa Cruz, CA) by repeated passage through a 27-gauge needle. The homogenate was centrifuged at 1000 × *g* for 15 min at 4 °C in a microcentrifuge to remove unbroken cells and nuclei. The postnuclear supernatant was centrifuged in an ultracentrifuge at 100,000 × *g* for 1 h at 4 °C in a Beckman TLA 100.2 rotor. The supernatant fraction was designated cytosol fraction, and the pellet was rinsed once with homogenization buffer and recentrifuged under the same conditions. The resulting pellet was solubilized in radioimmune precipitation assay buffer (150 mm NaCl; 1% Nonidet P-40; 0.25% sodium deoxycholate; 1 mm EDTA; 50 mm Tris-HCl (pH 7.4)) with protease inhibitors and a designated membrane fraction. Supernatant and pellet fractions containing the same volume of original cells were analyzed by SDS-PAGE and Western blotting.

##### SDS-PAGE and Western Blotting

Proteins were resolved on 6% SDS-PAGE and subsequently transferred to NitroPure nitrocellulose membrane (Micron Separations, Westborough, MA), and membranes were subjected to immunoblotting as previously described (71). The same NitroPure nitrocellulose was cut into sections and probed with anti-GBF1, anti-BIG2, anti-BIG1, anti-Golgin-97, anti-calnexin.

## RESULTS

### 

#### 

##### Depletion or Inactivation of GBF1 Prevents Membrane Recruitment of AP1 and GGA2, but Not Golgins, to the TGN

The function of GBF1 in the recruitment of the COPI coat is well documented, but the possible role of GBF1 in the recruitment of AP1 and the GGAs to TGN membranes is controversial. GBF1 has been shown to partially co-localize with GGA1–3 and AP1, and depletion of GBF1 from COS-7 cells caused relocation of GGA3, but not AP1, from TGN membranes (25). GBF1 has been shown to bind the β-subunit of the COPI coat and also the VHS-GAT domain of GGA1–3, suggesting that it may participate in the recruitment of both COPI and clathrin coats (25). However, treatment of Vero cells (derived from kidney epithelial cells extracted from an African green monkey) with the GBF1-specific inhibitor GCA has been shown not to affect membrane recruitment of AP1 or GGA3 (26). Thus, we elected to comprehensively investigate whether GBF1 affects recruitment of coat proteins to TGN membranes using multiple approaches.

**FIGURE 1.**
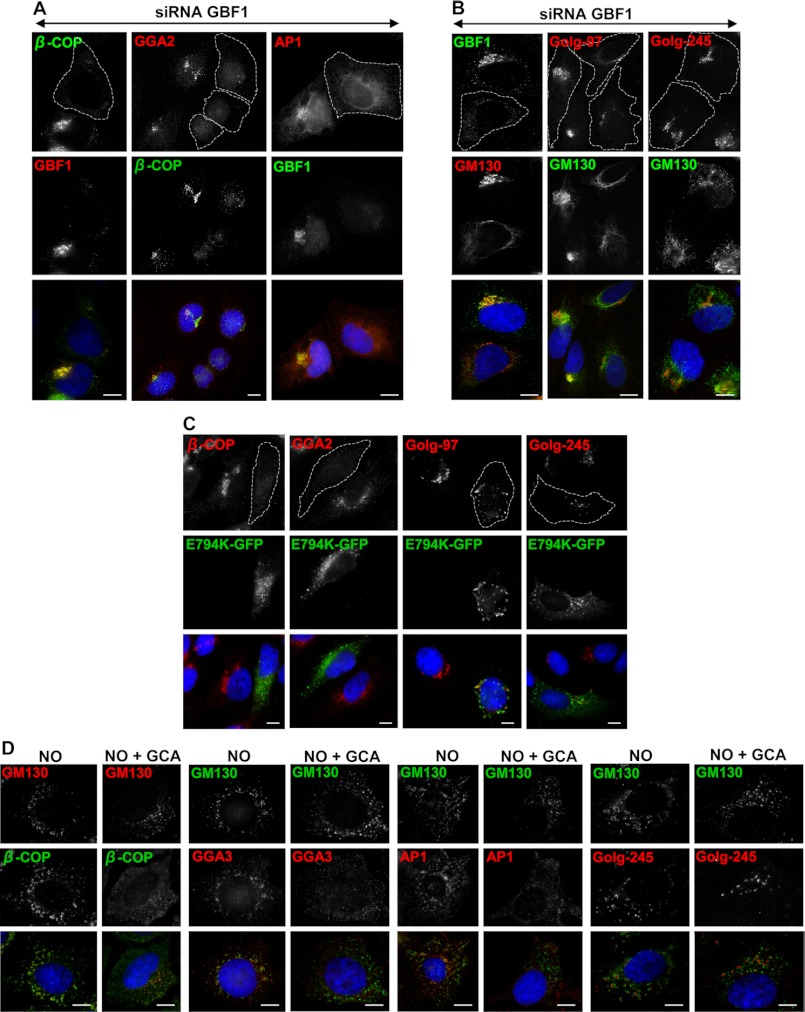
**GBF1 depletion or inactivation causes the dissociation of AP1, GGA2, and GGA3 from cellular membranes.** *A* and *B*, HeLa cells were transfected with siRNA oligos directed against GBF1, incubated for 72 h, and stained with indicated antibodies. *A*, GBF1 depletion blocks recruitment of β-COP, and the GGA2 and AP1 adaptors. *B*, GBF1 depletion causes fragmentation of the TGN but does not block recruitment of Golgin-97 and Golgin-245. *C*, HeLa cells were transfected with GBF1/E794K, incubated for 24 h, and stained with indicated antibodies. Expression of GBF1/E794K blocks membrane recruitment of β-COP and GGA2 and causes fragmentation of the TGN but does not block ARL-dependent recruitment of golgin-97 and golgin-245. *D*, HeLa cells were treated with NO alone or with NO and GCA, fixed, and stained with indicated antibodies. NO causes fragmentation of the Golgi and the TGN without affecting membrane recruitment of β-COP, GGA3, AP1, and Golgin-245. GCA causes the release of β-COP, GGA3, and AP1 from membranes without affecting the recruitment of Golgin-245. GBF1-depleted cells are *outlined. Bars* are 10 mm.

We first utilized siRNA to investigate the function of GBF1 in membrane recruitment of TGN clathrin adaptors. Transfection of HeLa cells with oligonucleotide duplexes complementary to sequences within human GBF1 resulted in a >80% reduction in the cellular levels of GBF1 after 3 days (see Fig. 3*A*). GBF1 depletion caused dissociation of the COPI coat, as shown by diffuse staining of the β-COP subunit (Fig. 1*A*), in agreement with previous reports (13, 14). Also consistent with previous observations, GBF1 depletion caused tubulation of the *cis*-Golgi, evident by staining with the *cis*-Golgi marker GM130 (Fig. 1*B*) (13). In all figures showing GBF1-depleted cells, we selected fields containing at least one non-depleted cell among the majority of depleted cells. Interestingly, depletion of GBF1 also caused dissociation of the GGA2 and AP1 coats, as evidenced by the loss of concentrated perinuclear staining (Fig. 1*A*). In contrast, recruitment of AP2, a related clathrin adaptor that is recruited to the plasma membrane and endosomes in a BFA-resistant manner, was normal in GBF1-depleted cells (data not shown).

One possible explanation for the loss of AP1 and GGA2 recruitment to the TGN in GBF1-depleted cells is loss of TGN membranes. To ensure that GBF1 depletion does not lead to complete disassembly of the TGN, we examined the localization of two TGN-resident golgins that contain a GRIP domain and are recruited through an Arl-mediated mechanism (for review, see Ref. 27). Golgin-97 and golgin-245 have been implicated in traffic from endosomes to the TGN, and loss of either protein prevents the transport of internalized Shiga toxin B from endosomes to the TGN (28). Importantly, depletion of GBF1 did not prevent membrane recruitment of golgin-97 and golgin-245, and both proteins were detected on membranous structures within the perinuclear area (Fig. 1*B*). In these images cells depleted of GBF1 are identified by tubulation of the GM130 Golgi marker, which consistently reflects efficient GBF1 depletion (13). In addition, GBF1 depletion had no effect on the recruitment of the EEA1 marker to early endosomes or endosomal architecture and distribution (data not shown). Together these observations suggest that TGN membranes may undergo partial fragmentation, but remain present, in cells depleted of GBF1.

An alternative means to explore the role of GBF1 in coat recruitment at the TGN is to utilize the dominant negative catalytically inactive GBF1/E794K mutant. Crystallographic analyses place the mutated glutamic acid residue within the active site of the Sec7 domain, and the E794K mutation has been shown to prevent GDP/GTP exchange on ARF bound to the Sec7 domain of a GEF (29–31). Expression of GBF1/E794K in HeLa cells containing endogenous GBF1 causes a dominant negative phenotype and results in the dissociation of the COPI coat (Fig. 1*C*) and the collapse of the Golgi (11). Therefore, we utilized the GBF1/E794K mutant to explore membrane recruitment of GGA2. Expression of GBF1/E794K led to loss of recruitment of GGA2 to TGN membranes (Fig. 1*C*). The effect is selective, as expression of GBF1/E794K had no effect on membrane recruitment of golgin-97 and golgin-245 (Fig. 1*C*). Although the TGN appears partially fragmented (consistent with the results see after siRNA-mediated depletion), the clearly detectable staining for golgin-97 and golgin-245 suggests that inactivation of GBF1 does not lead to a complete loss of TGN membranes.

To further confirm the role of GBF1 in the recruitment of TGN coats, we used the pharmacological inhibitor GCA. Like BFA, GCA is an interfacial inhibitor that binds to the same catalytic pocket formed by the Sec7 domain of a GEF and its ARF substrate. However, GCA is larger than BFA and can only fit into the GBF1-ARF catalytic pocket because only GBF1, but not BIG1 or BIG2, contains a three-amino acid (QNA) insertion within the binding interface that can accommodate the larger GCA (26). Treatment of cells with GCA caused the complete collapse of the Golgi and the complete dispersion of COPI, GGA2, and GGA3 (data not shown). However, the interpretation of these images is complicated due to possible fragmentation of membranes into structures too small to be resolved by light microscopy. Thus, to prevent membrane collapse and dispersion, cells were treated with nocodazole to prevent microtubule-mediated collapse of the Golgi into the ER. Nocodazole causes depolymerization of microtubules that leads to the fragmentation of the Golgi ribbon and the TGN into mini-stacks, as shown by the redistribution of GM130 into punctate structures (Fig. 1*D*). Importantly, nocodazole has no effect on the membrane association of COPI, GGA3, or AP1 (Fig. 1*D*). However, when nocodazole-treated cells were subsequently incubated with GCA, we observed dissociation of β-COP, GGA3, and AP1 (Fig. 1*D*). In contrast, GCA does not inhibit recruitment of golgin-97 (data not shown) and golgin-245 (Fig. 1*D*) to TGN membranes. We stress that the penetrance of the phenotypes was complete, *i.e.* all examined cells showed dissociation of β-COP, GGA3, or AP1, and all cells showed membrane-associated golgin-97 or golgin-245. Together, our results suggest that GBF1 activity is required for the recruitment of AP and GGA coats to the TGN.

##### GBF1 Activity Regulates the Localization of Proteins That Cycle between the TGN and Endosomes

The requirement for GBF1-mediated ARF activation in trafficking of proteins that cycle between the ER and Golgi has been well documented, and GBF1 depletion, expression of GBF1/E794K, and GCA treatment all cause mislocalization of Golgi proteins (11, 14, 26). AP1 and GGA1–3 have been implicated in the trafficking of proteins that cycle between the TGN and endosomes, and we explored the possible role of GBF1 in regulating the distribution of such proteins. TGN46 and the P-type ATPase ATP7A (also called Menkes protein, MNK) both localize to the TGN at steady state but cycle constitutively between the TGN, endosomes, and cell surface under basal conditions (32–34). TGN localization of TGN46 and MNK requires ARF activation, as BFA treatment and expression of the dominant inactive ARF1/N126I cause the dispersal of both proteins (35). Possible involvement of GBF1 in trafficking of these proteins has been suggested by the finding that expression of GBF1/E794K causes mislocalization of TGN46 and MNK (35).

**FIGURE 2.**
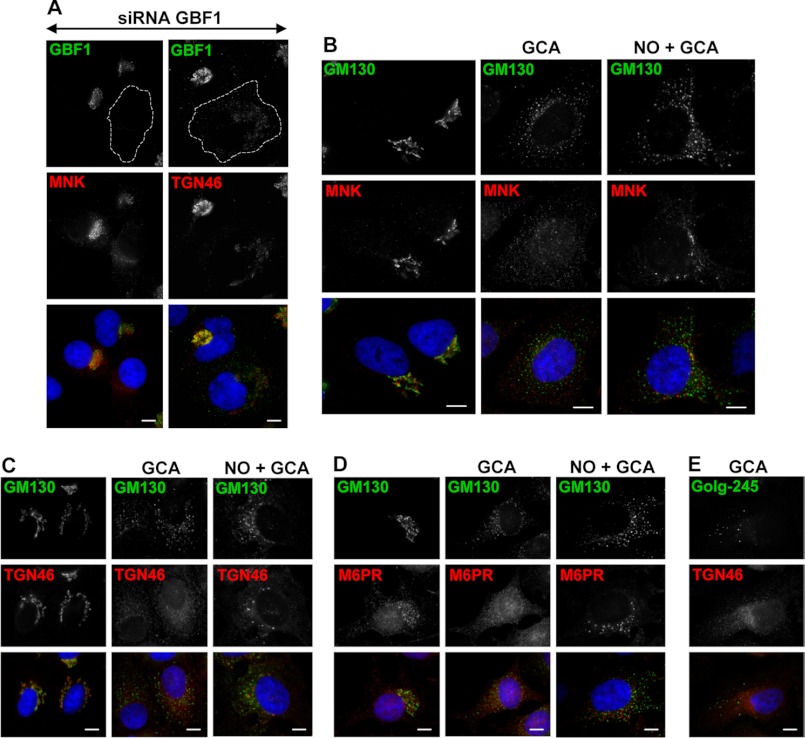
**GBF1 depletion or inactivation causes dispersal of TGN46, MNK, and M6PR.** *A*, HeLa cells were transfected with siRNA oligos directed against GBF1, incubated for 72 h, and stained with indicated antibodies. GBF1 depletion causes dispersion of MNK and TGN46. *B–E*, HeLa cells were mock-treated, treated with GCA alone, or treated with NO and GCA, fixed, and stained with the indicated antibodies. GCA causes complete dispersal of MNK, TGN46 and M6PR. Dispersal was limited in the presence of NO. GCA causes the dispersal of TGN46 while maintaining TGN elements containing golgin-245. *Bars* are 10 mm.

We further probed the possible function of GBF1 in TGN46 and MNK trafficking in GBF1-depleted cells and in cells treated with GCA. We show that the localization of TGN46 and MNK is disrupted in GBF1-depleted cells and that both of these proteins redistribute into perinuclear tubules and barely visible small elements scattered throughout the cell periphery (Fig. 2*A*). Similarly, treatment of cells with GCA causes complete dispersal of MNK (Fig. 2*B*), TGN46 (Fig. 2*C*), and M6PR (Fig. 2*D*). To examine whether this reflects extensive fragmentation of the membranes, cells were treated with nocodazole before GCA treatment. In nocodazole/GCA-treated cells, MNK (Fig. 2*B*), TGN46 (Fig. 2*C*), and M6PR (Fig. 2*D*) are clearly detected in punctate structures scattered throughout cells. Thus, GBF1-mediated ARF activation is required for proper cycling and TGN localization of MNK, TGN46, and M6PR. The dispersal of these proteins occurs despite the presence of TGN elements, as shown by the localization of golgin-245 in elements that lack TGN46 (Fig. 2*E*). Together, these observations suggest that GBF1 activity influences trafficking of TGN proteins, in agreement with the observed role for GBF1 in AP1 and GGA2 recruitment to TGN membranes.

##### GBF1 Is Required for Membrane Recruitment of BIG1 and BIG2

**FIGURE 3.**
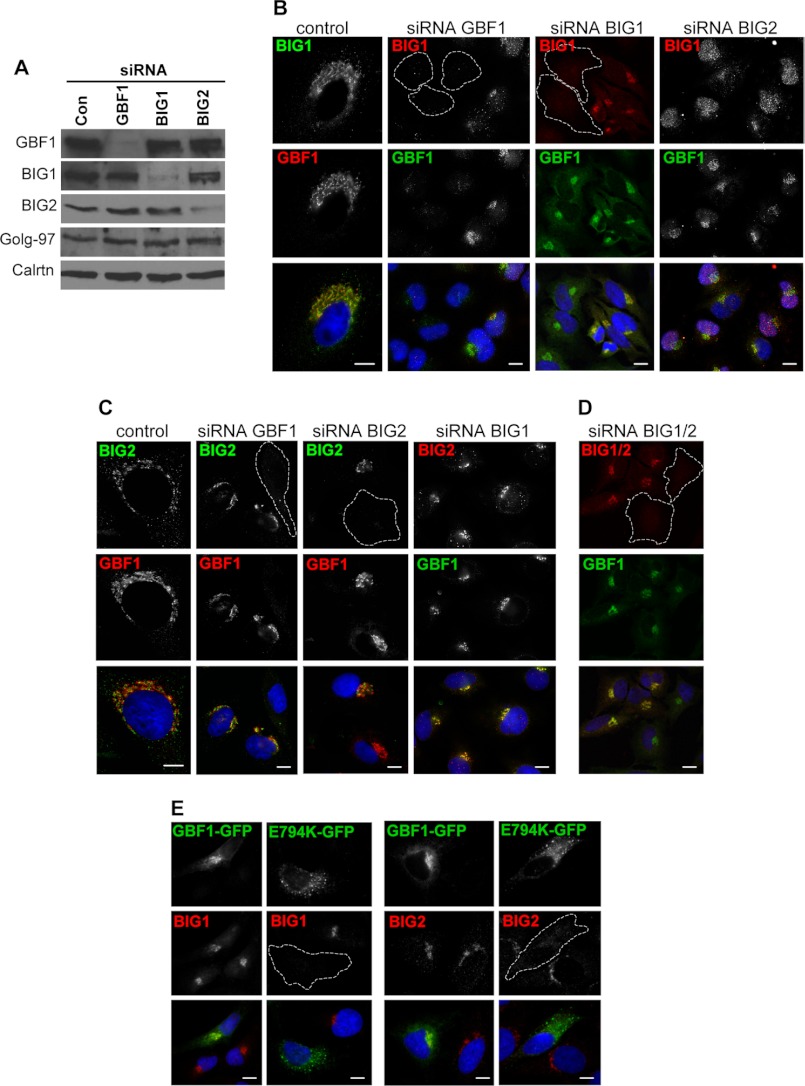
**GBF1 depletion or inactivation blocks recruitment of BIG1 and BIG2 to membranes.** *A–D*, HeLa cells were transfected with either control siRNA or siRNA oligos directed against GBF1, BIG1, or BIG2 for 72 h. *A*, control and depleted cells were lysed, and the lysates were Western-blotted using the indicated antibodies. Western blots show specific depletion of GBF1, BIG1, and BIG2. Golgin-97 and calreticulin are shown as loading controls. *B–D*, control and depleted cells were fixed and stained with the indicated antibodies. Depletion of GBF1 blocks recruitment of BIG1 and BIG2 to membranes. Depletion of BIG1 or BIG2 has no effect on localization of GBF1. BIG2 depletion leads to partial nuclear relocation of BIG1. Simultaneous depletion of BIG1 and BIG2 has no effect on recruitment of GBF1 to membranes. *E*, HeLa cells were transfected with GFP-tagged wild-type GBF1 (*GBF1*) or GBF1/E794K (*E794K*). After 24 h, cells were fixed and stained with the indicated antibodies. Expression of wild-type GBF1 has no effect on BIG1 and BIG2 recruitment to perinuclear elements. In contrast, expression of GBF1/E794K caused dissociation and dispersal of BIG1 and BIG2. *Bars* are 10 mm.

Simultaneous depletion of BIG1 and BIG2 causes dissociation of AP1 and GGA3 and inhibits trafficking of the mouse TGN46 homolog, TGN38, from the endosomes to the TGN (14, 15). Because we observed similar phenotypes in cells lacking GBF1 activity, this raised the possibility that GBF1 may influence BIG1/2 function. To define the functional relationships between the three GEFs, we used RNAi to deplete GBF1, BIG1, and BIG2 from HeLa cells. Transfection of HeLa cells with oligonucleotide duplexes complementary to sequences within human GBF1, BIG1, or BIG2 resulted in a >80% reduction in the cellular levels of each protein after 3 days (Fig. 3*A*). Importantly, depletion of any one GEF did not influence the expression levels of the other two GEFs. Similarly, depletion of any GEF did not affect the expression level of the TGN marker golgin-97 or the ER marker calreticulin (Fig. 3*A*).

Staining of GBF1-depleted cells with antibodies directed against BIG1 or BIG2 shows loss of BIG1 or BIG2 staining (Fig. 3, *B* and *C*), suggesting that GBF1 depletion blocks recruitment of BIGs to cellular membranes. In contrast, depletion of either BIG1 (Fig. 3*B*) or BIG2 (Fig. 3*C*) had no effect on the localization of GBF1. The distribution of GBF1 in cells depleted of BIG1 or BIG2 is analogous to that in control cells, suggesting that the architecture of the Golgi is not affected by BIG1 or BIG2 depletion. This observation is in agreement with previous reports that simultaneous depletion of BIG1 and BIG2 has no effect on membrane recruitment of COPI or Golgi morphology (14, 36).

Depletion of BIG1 had no effect on membrane recruitment of BIG2 (Fig. 3*C*), and depletion of BIG2 had no effect on membrane recruitment of BIG1 (Fig. 3*B*), in agreement with previous reports (15, 37). However, BIG2 depletion did lead to partial relocalization of BIG1 into the nucleus. BIG1 predominantly localizes to the perinuclear region of cells but has also been detected within the nucleus, especially under conditions of serum starvation or high cAMP levels (37, 38). Importantly, co-depletion of BIG1 and BIG2 did not affect membrane recruitment of GBF1 (Fig. 3*D*).

Loss of GBF1 could lead to loss of BIGs recruitment either through loss of GBF1-mediated ARF activation or due to loss of important protein-protein interactions. To explore the role of GBF1-catalyzed ARF activation in membrane recruitment of BIG1 and BIG2, HeLa cells were transfected with the catalytically inactive mutant GBF1/E794K. Cells expressing GBF1/E794K show diffuse staining for BIG1 and BIG2 (Fig. 3*E*), suggesting that GBF1-mediated ARF activation is required for BIG1 and BIG2 recruitment to membranes.

##### BFA and GCA Have Distinct Effects on Membrane Recruitment of BIG1 and BIG2

**FIGURE 4.**
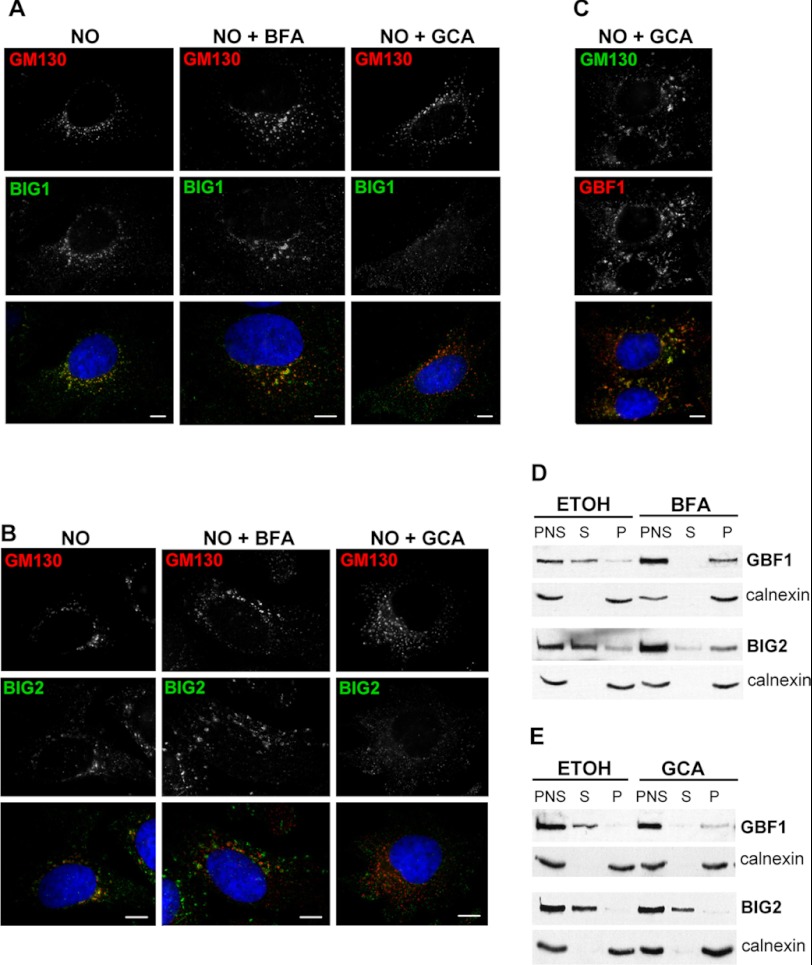
**Effect of BFA and GCA on membrane association of BIG1 and BIG2.** *A–C*, HeLa cells were treated with NO alone, with NO and BFA, or with NO and GCA, fixed, and stained with indicated antibodies. NO caused fragmentation of the Golgi and TGN. BIG1 and BIG2 remained on membranes in the presence of BFA but dissociated from membranes in the presence of GCA. In contrast, GBF1 remained on membranes in GCA-treated cells. *D* and *E*, HeLa cells were mock-treated with ethanol (ETOH) or with BFA or GCA and fractionated into post-nuclear supernatant (*PNS*), which was subsequently separated into supernatant containing cytosolic proteins (*S*) and membrane pellet (*P*). Equivalent amounts of cytosol and pellet fractions were analyzed by SDS-PAGE and blotted with indicated antibodies. In control cells, GBF1 and BIG2 are largely cytosolic. In the presence of BFA, both GBF1 and BIG2 translocated to membranes. In contrast, GCA caused translocation of GBF1 to membranes but caused dissociation of BIG2 from membranes.

To further explore the role of GBF1 in membrane recruitment of BIG1 and BIG2, we compared the effects of BFA (a general ARFGEF inhibitor that affects both GBF1 and the BIGs) and GCA (a selective GBF1 inhibitor) on membrane association of these GEFs. In these experiments cells were first treated with nocodazole to inhibit the complete collapse of the Golgi into the ER and then with either BFA or GCA. Nocodazole caused the formation of Golgi mini-stacks but had no effect on membrane association of either BIG1 (Fig. 4*A*) or BIG2 (Fig. 4*B*). Importantly, subsequent treatment with BFA also maintained membrane recruitment of BIG1 and BIG2. This is consistent with the expected stabilization of all three ARFGEFs in abortive GEF-ARF·GDP-BFA complexes (39–41). BFA has been shown to stabilize GBF1 on membranes when assessed by FRAP (42–44). Similarly, BIG1 (9) and BIG2 (45) have been shown to remain membrane-associated in cells treated with BFA. In contrast, treatment of cells with the selective GBF1 inhibitor GCA led to decreased association of BIG1 (Fig. 4*A*) and BIG2 (Fig. 4*B*) with membranes. We stress that dissociation of BIG1/2 was observed in all examined cells. GCA treatment had no effect on membrane recruitment of GBF1 (Fig. 4*C*), as expected as GBF1 is predicted to bind GCA and be stabilized in an abortive complex via a mechanism analogous to that shown for BFA (41).

The differential effects of BFA and GCA on membrane association of GBF1 and BIG2 were confirmed by biochemical fractionation experiments. Cells were mock-treated or treated with either BFA or GCA followed by separation into cytosol and total membrane fractions. The levels of GBF1 and BIG2 in each fraction were assessed by Western blotting. In mock-treated cells, GBF1 and BIG2 were predominantly recovered in the cytosolic fraction, consistent with the constant cycling of these proteins between the membrane and the cytosol (Fig. 4*D*). BFA treatment caused a significant shift of GBF1 and BIG2 to membranes (Fig. 4*D*), as expected by the generation of a stabilized GEF-ARF·GDP-BFA complex. In contrast, GCA treatment led to increased membrane association of GBF1 but not of BIG2 (Fig. 4*E*). The lack of BIG2 recruitment is consistent with the immunofluorescence data and confirms that GBF1-mediated ARF activation is required for membrane association of BIG2.

##### Active Forms of ARF4 and ARF5 Regulate Membrane Recruitment of BIG1 and BIG2

GBF1 activity appears required for BIG1 and BIG2 association with membranes, suggesting that ARFs activated by GBF1 might facilitate binding of BIGs to membranes. GBF1 has been shown to minimally activate a mixture of ARF1 and ARF3 and to have high exchange activity on ARF5 *in vitro* (46). In addition, *in vivo* studies showing GBF1 co-precipitating with ARF1 and ARF4, but not ARF3, suggest that GBF1 may use ARF1 and ARF4 as substrates (13). In agreement, ARF3 shows low levels of co-localization with ERGIC53 (47), whereas ARF4 and ARF5 co-localize extensively with ERGIC53 and GBF1 (9). Simultaneous depletion of ARF1 and ARF4 causes dissociation of COPI and induces Golgi tubulation (48), a phenotype analogous to that observed in cells depleted of GBF1 (13), implicating ARF4 as a likely GBF1 substrate. Importantly, simultaneous depletion of ARF1 and ARF3 has no effect on the Golgi (48), in agreement with minimal activity of GBF1 on ARF3.

**FIGURE 5.**
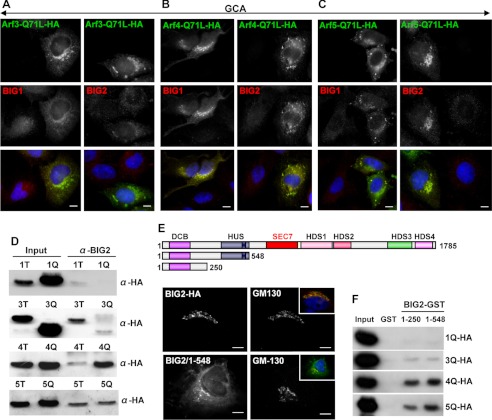
**Active ARF4 and ARF5 mediate membrane recruitment of BIG1 and BIG2.** *A—C*, HeLa cells were transfected with HA-tagged dominant active (Q71L) forms of ARF3 (*A*), ARF4 (*B*), and ARF5 (*C*) for 24 h. Cells were then treated with GCA, fixed, and stained with the indicated antibodies. Transfection with activated ARF4 and ARF5 cause association of BIG1 and BIG2 with membranes in GCA-treated cells. Expression of ARF3 does not rescue GCA-mediated dissociation of BIG1 and BIG2 from membranes. *Bars* are 10 mm. *D*, HeLa cells were transfected with HA-tagged inactive (T31N) or constitutively active (Q71L) forms of ARF1, ARF3, ARF4, and ARF5 for 24 h, and BIG2 was immunoprecipitated from cell lysates. The starting lysate (input) and the bound material were immunoblotted with anti-HA antibodies. Significant levels of active ARF4 and ARF5 but not ARF1 or ARF3 bind to BIG2. *E*, structural motifs within BIG2 and the BIG2/1–548 and the BIG2/1–250 constructs are shown. *HDS*, homology downstream of Sec7. HeLa cells were transfected with HA-tagged full-length BIG2 or BIG2/1–548 for 24 h, fixed, and stained with anti-HA antibodies. BIG2/1–548 targeted to the TGN. *F*, HeLa cells were transfected with HA-tagged constitutively active (Q71L) forms of ARF1, ARF3, ARF4, and ARF5 for 24 h and lysed. Lysates were incubated with purified GST or GST chimeras containing DCB+HUS (amino acids 1–548) or DCB (amino acids 1–250) domains of BIG2. The starting lysate (input) and the bound material were immunoblotted with anti-HA antibodies. Active ARF4 and ARF5, but not ARF1 or ARF3, bound to the DCB+HUS and the DCB only domain but not to GST.

If GBF1-mediated activation of ARF4 and ARF5 facilitates BIG1 and BIG2 recruitment to the TGN, then BIG1 and BIG2 should be recruited to membranes in cells expressing activated ARF4 or ARF5 even when GBF1 is inactivated. In contrast, active ARF3 should not facilitate BIG1 and BIG2 recruitment. Thus, we examined membrane recruitment of BIG1 and BIG2 in HeLa cells transfected with the active (Q71L) forms of ARF3, ARF4, or ARF5 and treated with GCA to inactivate GBF1. In cells expressing active ARF3 and treated with GCA, the majority of BIG1 and BIG2 is dissociated from membranes (Fig. 5*A*). In contrast, expression of active ARF4 (Fig. 5*B*) and ARF5 (Fig. 5*C*) resulted in increased recruitment of BIG1 and BIG2 to membranes in comparison to surrounding untransfected cells. These observations suggest that GBF1-mediated activation of ARF4 and ARF5 facilitates membrane recruitment of BIG1 and BIG2.

##### Active Forms of ARF4 and ARF5 Interact with the N-terminal Domain of BIG2

To assess whether active forms of ARF4 and ARF5 might interact with BIGs, we assessed the level of co-immunoprecipitation between endogenous BIG2 and exogenously expressed active Q71L or inactive T31N mutants of ARF4 and ARF5. As shown in Fig. 5*D*, BIG2 co-precipitated with the active ARF4/Q71L but not the inactive ARF4/T31N mutant, suggesting that activated ARF4 may generate membrane binding sites for BIG2. BIG2 also co-precipitated with the active ARF5/Q71L mutant, indicating that activated ARF5 also might facilitate BIG2 recruitment to membranes. Interestingly, BIG2 bound significant amounts of ARF5/T31N, raising the possibility that ARF5 might be a BIG2 substrate in cells. BIG2 co-precipitated with the inactive ARF1/T31N but not the active ARF1/Q71L mutant. Similarly, BIG2 preferentially co-precipitated with the inactive ARF3/T31N mutant but showed minimal interaction with the active ARF3/Q71L. BIG2 is expected to bind the inactive ARF1/T31N and ARF3/T31N because ARF1 and ARF3 in the GDP-bound form have been reported to function as BIG2 substrates (49, 50).

The preferential recovery of active forms of ARF4 and ARF5 with BIG2 is consistent with the notion that these ARFs might recruit BIG2 to TGN membranes. Previous studies showed that the N-terminal region of BIG2 (amino acids 1–552) is sufficient to target BIG2 to the TGN (9). Thus, if active forms of ARF4 and ARF5 facilitate BIG2 recruitment to membranes, they would be expected to interact with the N-terminal region of BIG2. We generated a slightly smaller BIG2/1–548 fragment containing the dimerization and cyclophilin binding (DCB) and the homology upstream of Sec7 (HUS) domains and first showed that it also targets to the TGN when expressed in HeLa cells (Fig. 5*E*). We then generated an analogous construct as a GST chimera and assessed its ability to interact with activated ARF4 and ARF5 in pulldown assays. As controls we also assessed interaction of the GST-BIG2/1–548 fragment with active forms of ARF1 and ARF3. As shown in Fig. 5*F*, we detect robust binding of ARF4/Q71L and ARF5/Q71L to the GST-BIG2/1–548 chimera. In contrast, the chimera binds minimal amounts of ARF1/Q71L and ARF3/Q71L. GST was used as a control and does not bind any of the ARFs. Our results indicate that active forms of ARF4 and ARF5 interact with the N-terminal domain of BIG2, the same region that is sufficient to target BIG2 to the TGN. Furthermore, a construct containing only the N-terminal DCB domain (amino acids 1–250) of BIG2 also bound ARF4/Q71L and ARF5/Q71L but not ARF1/Q71L or ARF3/Q71L, suggesting that the interaction between BIG2 and ARF4/5 might be mediated by the DCB domain.

##### GBF1 Localizes to Pre-Golgi, Golgi, and TGN Compartments

The association of BIG1 and BIG2 with the TGN appears to be mediated through active forms of ARF4 and ARF5, two ARFs thought to be activated by GBF1. However, multiple immunofluorescence localization studies showed ARF4, ARF5, and GBF1 to be concentrated in the early compartments of the ER-Golgi interface and the Golgi (10, 11, 46, 51). Whether GBF1 also may localize to additional compartments and activate ARFs therein is less certain. That GBF1 may have a wider distribution is suggested by the finding that GBF1 partially co-localizes and co-precipitates with the TGN adaptors GGA1–3 (25). The interaction involves the GAT domain of GGAs and the N-terminal region of GBF1 and may be direct as it can be detected in a yeast two-hybrid system (25).

**FIGURE 6.**
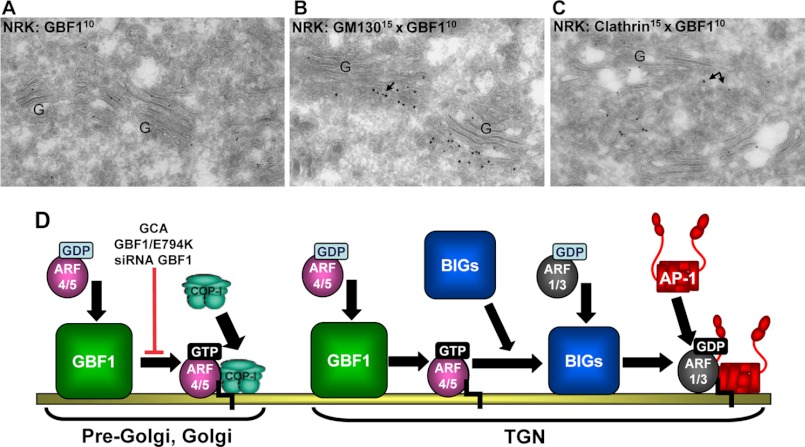
**Localization of GBF1 within the secretory pathway.** *A–C*, NRK cells were labeled with anti-GBF1 (small gold (*G*)) (*A*) and either anti-GM130 (*B*) or anti-clathrin (*C*) antibodies (large gold). GBF1 was detected throughout the Golgi stack and localized to early compartments as defined by the *cis*-Golgi marker GM130 and to late compartments defined by the TGN marker clathrin. Quantitation of GBF1 distribution is presented in Table 1. *D*, shown is a model for GBF1 function in the secretory pathway. In pre-Golgi, Golgi, and the TGN GBF1 catalyzes GDP/GTP exchange on ARF4/ARF5. Activation of these ARFs can be inhibited by GBF1 siRNA, expression of GBF1/E794K, BFA, or GCA. At the pre-Golgi and Golgi the activated ARFs regulate recruitment of COPI to pre-Golgi and Golgi membranes. At the TGN, the activated ARFs mediate recruitment of BIG1 and BIG2 to TGN membranes. Membrane-associated BIG1 and BIG2 then activate their substrates ARF1/ARF3, resulting in membrane recruitment of TGN coats, such as AP1 and the GGAs. We propose that GBF1 acts as a master regulator of COPI and clathrin-mediated coating events in the secretory pathway.

To explore whether GBF1 might mediate ARF activation at the TGN, we analyzed GBF1 localization at the ultrastructural level. We used thin frozen sections of NRK cells and performed double label immunogold labeling of endogenous GBF1 relative to the *cis*-Golgi marker GM130 and the TGN marker clathrin to quantitate GBF1 distribution (63). We found that GBF1 is broadly distributed within the Golgi region (Fig. 6*A*). GBF1 is clearly detected within early pre-Golgi and *cis*-Golgi compartments labeled with GM130 (Fig. 6*B*) in agreement with the light level localization. However, GBF1 and is also detected at the TGN compartments that label with clathrin (Fig. 6*C*). Quantification indicates that GBF1 predominantly localizes to the *cis*-most Golgi cisterna (∼22% of total gold particles) but is also detected at significant levels (∼9% of total gold particles) at the TGN (Table 1). Thus, GBF1 is localized at the TGN and can mediate ARF activation therein to facilitate BIG1 and BIG2 recruitment to TGN membranes.

**TABLE 1:** **GBF1 localization within pre-Golgi, Golgi, and the TGN** Gold particles were counted in pre-Golgi compartments (membranes found in an area up to 400 nm at the *cis* side of the Golgi), cisterna 1–5 (C1 being *cis* and C5 being *trans*), the TGN (membranes found in the an up to 400 nm at the *trans* side of the Golgi), and the peri-Golgi/rims of cisterna. Percentages of total gold particles found over the indicated categories ±S.E. were calculated. Data were based on sampling of 10 individual Golgi from 6 different EM grids; 3 that were double-immunogold-labeled for GM130 and GBF1- and -3-labeled for clathrin and GBF1 (analogous to the images shown in Fig. 6, *B* and *C*). This approach was taken to avoid any bias toward sampling for the *cis* or *trans* side of the Golgi. Percentages were calculated by determining per grid the relative distribution of the gold particles. These percentages were added up, and the average percentage ±S.E. was calculated. Total number of Golgi counted: 60. Total number of gold particles counted: 320.

	Pre-Golgi	C1	C2	C3	C4	C5	TGN	Peri-Golgi/rims
Average % of gold particles	15.8	22.1	17.6	13.9	10.3	5.3	9.0	5.9
S.E.	2.07	2.26	1.45	3.03	0.48	2.10	2.84	2.02

## DISCUSSION

Protein transport within the secretory and endocytic pathways is dependent on coating events that facilitate cargo movement between distinct compartments. Coats select and concentrate cargo proteins into nascent buds and facilitate membrane deformation before vesicle budding. Within the Golgi, protein traffic is orchestrated by the heptameric COPI coat and the adaptor-clathrin coats that include the multimeric AP1 and the monomeric GGAs (for review, see Ref. 18). Both types of coats are recruited to membranes by activated (GTP-bound) forms of ARFs. Three large BFA-sensitive GEFs, GBF1, BIG1, and the closely related BIG2, appear to regulate ARF activation required for coating events at the Golgi.

The role of GBF1 in maintaining the architecture of the Golgi and facilitating protein traffic at the ER-Golgi interface is well documented (13, 14, 26, 44). GBF1 localizes to early secretory compartments, including the ERGIC and early Golgi, where it extensively co-localizes with COPI (9–11). GBF1 appears to be the only cellular GEF whose ARF activating function supports COPI recruitment (10, 13, 14). This is suggested by the finding that only overexpression of GBF1, but not BIG1/BIG2, protects cells from BFA-induced dissociation of COPI, and only depletion or inactivation of GBF1 causes loss of COPI from membranes and results in extensive tubulation and eventual collapse of the Golgi into the ER. In contrast, the removal of BIG1, BIG2, or both BIG1/2 have minimal effect on Golgi architecture, ER-Golgi trafficking, or COPI recruitment (14, 15).

The precise roles BIG1 and BIG2 play at the TGN remain controversial. Both BIG1 and BIG2 localize mainly to the TGN and show extensive overlap with clathrin but do not co-localize with COPI (9, 21, 22, 45, 52, 53). BIG2 appears to have a wider localization and also is detected on recycling endosomes (23, 24). Although some studies suggest that BIG1 and BIG2 have redundant functions, other reports showcase distinct roles for BIG1 and BIG2. BIG1 cannot compensate for lack of BIG2 function in mammals, as mutations in BIG2 lead to the neurological disease microcephaly with periventricular heterotopia in humans (54) and deletion of the mouse BIG2 (Arfgef2) gene causes early embryonic lethality (55). In support of non-redundant functions, studies in cells in culture uncovered distinct phenotypes caused by BIG1 and BIG2 depletions. Depletion of only BIG1 leads to a “looser” Golgi and has no effect on recycling endosomes, whereas depletion of BIG2 has no effect on Golgi architecture but causes changes in the architecture and function of recycling endosomes (15, 56). In addition, depletion of BIG1, but not BIG2, dramatically inhibits the reorientation of the Golgi toward the protruding edge of the cell during wound healing (58). However, BIG1 and BIG2 may also control similar events and partially compensate for each other in some processes. This is suggested by the finding that the depletion of only BIG1 or only BIG2 has no visible effect on the localization of TGN proteins such as furin, TGN46, M6PR, and sortilin, which cycle between the TGN and endosomal compartments, whereas the simultaneous depletion of both BIG1 and BIG2 causes the redistribution of such proteins (14, 15). Similarly, although depletion of only BIG1 or only BIG2 causes a small reduction in the localization of AP1 to the TGN, the simultaneous depletion of both BIG1 and BIG2 causes the disappearance of AP1 from the TGN (15). Thus, both BIG1 and BIG2 appear to regulate ARF activation that facilitates the recruitment of clathrin adaptors to the TGN. In agreement, overexpression of either BIG1 or BIG2 can protect cells from BFA-mediated dissociation of AP1 coat (14). The role of BIG1 and BIG2 in the recruitment of GGAs appears controversial; although Ishizaki *et al.* (14, 15) reported that BIG1/2 depletion does not affect GGA3 localization, Manolea *et al.* (14, 15) showed the disappearance of GGA3 from the TGN from cells lacking both BIG1 and BIG2. The reason for this disparity is currently unknown.

Thus, it appears that GBF1 regulates exclusively COPI coating events, whereas the BIGs preferentially facilitate clathrin-mediated coating events. However, the finding that GBF1 directly interacts with GGAs and that GBF1 depletion affects the localization of TGN coats (25) and causes relocation of TGN46 (14) suggested a possible interplay between the coating events, raising the possibility that GBF1 may also regulate clathin-mediated coating. Unexpectedly, we discovered that depletion of GBF1, expression of the catalytically inactive GBF1/E794K mutant, or treatment of cells with GCA inhibits the recruitment of BIG1/2 to TGN membranes, suggesting that TGN recruitment of BIG1 and BIG2 requires GBF1 activity. The consequences of BIG1/2 dissociation in GBF1-compromised cells parallel those seen in cells simultaneously depleted of BIG1 and BIG2. Specifically, GBF1-depleted cells show the dissociation of AP1 and GGA coats and the redistribution of TGN46, M6PR, and MNK (proteins that cycle between the TGN and endosomal compartments) from their normal perinuclear localization. Importantly, GBF1 depletion did not cause the collapse of the TGN or dissociation of the TGN marker golgin-245, which is recruited to membranes through an Arl-dependent mechanism (59, 60). This is in agreement with previous findings showing that the localization of golgin-245 was unaffected by BIG1/2 depletion (15), indicating that an intact TGN compartment persists despite the absence of BIG1/2-mediated ARF activation. Thus, GBF1 affects the localization and thereby the function of BIG1/2.

We explored the possible mechanism through which GBF1 regulates BIG1/2 recruitment by considering that GBF1-activated ARFs may regulate BIG1/2 recruitment. GBF1 has been shown to preferentially catalyze GDP/GTP exchange on ARF5 *in vitro* (46) and to co-precipitate with ARF4 from cell lysates (13), suggesting that these class II ARFs are its substrates. In support, ARF4 and ARF5, but not ARF3, extensively co-localize with GBF1 at the ERGIC and the Golgi (61, 62). We found that active forms of ARF4 and ARF5 (but not ARF3) restored BIG1 and BIG2 recruitment to the TGN in the absence of GBF1 activity. Our findings suggested a GEF/ARF/GEF cascade model in which GBF1 activates ARFs that subsequently facilitate BIG1/2 recruitment to the TGN, which subsequently activate ARFs that recruit clathrin adaptors. In support of his model, only the active, but not the inactive forms of ARF4 and ARF5, co-precipitate with endogenous BIG2, indicating that complexes of active ARF4/5 and BIG2 exist in cells. The association of BIG1 (21) and BIG2 (9) with the TGN has been shown to be mediated by the N-terminal region containing the DCB ad HUS domains proximal to the catalytic Sec7 domain. Our model that activated ARF4/5 recruit BIGs to membranes predicts that activated ARF4/5 (but not ARF3) should interact with the N-terminal region of the BIGs. In support, we detected an interaction of activated ARF4/5 (but not ARF3) with the N-terminal (amino acids 1–548) fragment of BIG2 containing the DCB and HUS domains. These binding experiments, which included co-precipitations from cell lysates and pulldown assays of exogenously expressed ARFs from cell lysates using recombinant N-terminal domain of BIG2, do not distinguish between the possibility that ARF4/5 directly bind BIG1/2 or that additional components may act as molecular linkers. In the first scenario, BIGs would directly bind the activated ARFs and act as ARF effectors, whereas in the latter case, unknown ARF effectors would bind BIGs to recruit them to membranes.

Our model of GBF1-mediated BIG1/2 recruitment to the TGN necessitates the localization of GBF1 to the TGN. This at first appeared improbable, as previous immunofluorescence studies indicate that GBF1 is concentrated within pre-Golgi and early Golgi compartments, where it co-localizes with ERGIC markers such as ERGIC-58 and *cis*-Golgi markers such as GM130 (10, 11, 46). However, other immunofluorescence studies detected GBF1 in the TGN, where it co-localized with TGN adaptors (25). Thus, we explored GBF1 localization at the electron microscope level within the pre-Golgi/Golgi/TGN continuum. Our ultrastructural analysis indicates that although GBF1 is most abundant in the *cis*-Golgi, in agreement with immunofluorescence results, GBF1 also localizes to more distal Golgi cisterna and is present in significant amounts (∼41% of the level detected in the *cis*-cisterna) at the TGN. Thus, GBF1 localizes at the TGN where it can catalyze activation of ARF4/5 that subsequently can facilitate the recruitment of BIG1 and BIG2 to TGN membranes.

GBF1 is a soluble protein that rapidly cycles between cytosolic and membrane-associated pools in a process that is at least partially regulated by its catalytic activity (42, 43). Theoretically, GBF1 has full access to all the compartments of the secretory and endocytic pathways, yet shows strong selective localization to only a subset of these membranes. Our understanding of the mechanisms that may recruit GBF1 to distinct membranes is limited. Rab1 appears to facilitate GBF1 recruitment to pre-Golgi ERGIC and early Golgi compartments (12, 64), but it is unlikely that Rab1 is involved in GBF1 recruitment to the TGN, as Rab1 localization appears restricted to compartments at the ER-Golgi interface (65, 66). Perhaps a TGN-localized Rab, like Rab6 (67, 68), may participate in GBF1 recruitment at that site. It is also possible that distinct mechanisms and components regulate association with different membranes. Our results present an additional interesting conundrum; How can GBF1-activated ARF4/5 recruit different effectors in the early *versus* late Golgi; *i.e.* what keeps GBF1-activated ARF4/5 from recruiting the BIGs to the *cis*-Golgi and what prevents GBF1-activated ARF4/5 from recruiting COPI to the TGN? The most plausible explanation is that additional factors participate in the recruitment process and that those factors are compartment-specific. Defining the full interactome of GEFs and ARFs and assessing the localization of each interactor might provide insight into compartment-specific recruitment mechanisms. The temporal and spatial regulation of GEF recruitment is critical for cargo transport as it initiates the budding event, and elucidating the upstream signaling pathways that lead to correct GEF positioning is necessary to understand vesicular trafficking.

Our findings underscore the complexity of GEF recruitment mechanisms, as multiple parameters that regulate GEF recruitment have been identified. For GBF1, interactions with Rab1 (12, 64) and binding to the ARF substrate (42, 43) appear to regulate residency on Golgi membranes. Recruitment of BIG1/2 also seems to be complex and to involve multiple binding partners. In addition to our findings invoking ARF4/5 in BIG1/2 recruitment *in vitro* and *in vivo*, studies with the fly *Drosophila melanogaster* homologue of BIG1/2 called Sec71 implicated the small GTPase Arl1 in Sec71 binding to membranes *in vitro* (69). Correlative *in vivo* studies in mammalian cells showed that depletion of Arl1 or overexpression of Arl1 effectors prevents localization of BIG1/2 to the TGN, suggesting that activated Arl1 is required for BIG1/2 recruitment to the TGN. Thus, it appears that activated members of the Arf (ARF4/5) and Arl (Arl1) families of small GTPases facilitate recruitment of BIG1/2 to the TGN. Interestingly, both Arl1 and ARF4/5 interact with the N-terminal DCB+HUS region of Sec71 or BIG1/2, respectively. The binding sites for Arl and ARFs within the DCB+HUS region are unknown, and it is possible that both GTPases must engage the GEF simultaneously in a coincidence detector mechanism to facilitate membrane recruitment.

A distinct mechanism appears to regulate the recruitment of the yeast homologue of BIG1/2, called Sec7. In this case the highly conserved domain downstream of the Sec7d (HDS1) was shown to bind activated ARF1 and to be required for membrane association (70). Sec7 lacking the HDS1 domain but containing the N-terminal DCB+HUS+Sec7 region does not bind activated ARF1 and does not associate with membranes. Thus, in yeast it is the HDS1 region that recruits Sec7 to the yeast TGN rather than the DCB+HUS region (that binds Arl1 and ARF4/5) that recruits BIG1/2 to membranes in mammalian cells. Thus, it is possible that the machinery for recruiting GEFs to the TGN in yeast and mammalian cells operates through different mechanisms. Interestingly, although yeast appear to have an Arl1 homologue, they only have class I ARFs (the yeast ARF1 and ARF2 most likely arose from a gene duplication) and do not have class II ARFs (ARF4 and ARF5), suggesting a possibly divergent evolution of recruitment mechanisms (57).

The significance of our findings is that they suggest a novel GEF/ARF/GEF cascade in which ARFs activated by one GEF (GBF1) facilitate the recruitment of other GEFs (BIG1/2). This sequential order of events initiates with GBF1 activating ARF4 and ARF5, which then recruit BIG1 and BIG2, which subsequently activate ARF1 and ARF3. In this model, the initial GEF has two roles; 1) it generates a set of activated ARFs that directly recruits a set of effectors and 2) facilitates recruitment of other GEFs to generate a distinct set of activated ARFs that recruits a distinct set of effectors. Thus, within the pre-Golgi and the Golgi, GBF1 catalyzes GDP/GTP exchange on its ARF substrates, which then facilitate recruitment of the COPI coatomer complex to pre-Golgi and Golgi membranes. However, at the TGN, GBF1 initiates an ARF/GEF cascade by activating ARF4/5 that then mediates the recruitment of BIG1 and BIG2 to the TGN. Membrane-bound BIG1 and BIG2 then activate their ARF substrates, ARF1 and ARF3, which in turn mediate the recruitment of TGN coats such as AP1 and GGA1–3. The dual role of GBF1 within the secretory pathway may function as an integrator to regulate both COPI and clathrin-mediated coating events within the entire pathway. Potentially, the cascade may act as a mechanism for coupling the functions of multiple GEFs to simultaneously coordinate organelle biogenesis and sequential trafficking of cargo proteins.

## Footnotes

3 The abbreviations used are:

ARF: ADP ribosylation factor
GEF: guanine nucleotide exchange factor
AP1: adaptor protein 1
BFA: brefeldin A
COP: coat protein complex
ER: endoplasmic reticulum
GCA: golgicide A
GGA: gamma-ear containing Golgi-localized adaptor
M6PR: mannose-6-phosphate receptor
MNK: Menkes protein (ATP7A)
TGN46: *trans*-Golgi protein of *M*_r_ 46
ERGIC: ER-Golgi intermediate compartment
DCB: dimerization and cyclophilin binding
HUS: homology upstream of Sec7
NO: nocodazole.
